# Using synchrotron radiation angiography with a highly sensitive detector to identify impaired peripheral perfusion in rat pulmonary emphysema

**DOI:** 10.1107/S090904951300040X

**Published:** 2013-02-08

**Authors:** Hiromichi Ito, Shonosuke Matsushita, Kazuyuki Hyodo, Yukio Sato, Yuzuru Sakakibara

**Affiliations:** a Graduate School of Comprehensive Human Science, Department of Thoracic and Cardiovascular Surgery, University of Tsukuba, 1-1-1 Tennodai, Tsukuba, Ibaraki 305-8575, Japan; b Faculty of Health Science, Tsukuba University of Technology, Tsukuba, Ibaraki 305-8521, Japan; c High Energy Accelerator Research Organization, KEK, Tsukuba, Ibaraki 305-0801, Japan

**Keywords:** pulmonary emphysema, pulmonary microcirculation, synchrotron radiation angiography, sensitivity, HARP detector

## Abstract

Owing to limitations in spatial resolution and sensitivity, it is difficult for conventional angiography to detect minute changes of perfusion in diffuse lung diseases, including pulmonary emphysema (PE). However, a high-gain avalanche rushing amorphous photoconductor (HARP) detector can give high sensitivity to synchrotron radiation (SR) angiography. SR angiography with a HARP detector provides high spatial resolution and sensitivity in addition to time resolution owing to its angiographic nature. The purpose of this study was to investigate whether this SR angiography with a HARP detector could evaluate altered microcirculation in PE. Two groups of rats were used: group PE and group C (control). Transvenous SR angiography with a HARP detector was performed and histopathological findings were compared. Peak density of contrast material in peripheral lung was lower in group PE than group C (*p* < 0.01). The slope of the linear regression line in scattering diagrams was also lower in group PE than C (*p* < 0.05). The correlation between the slope and extent of PE in histopathology showed significant negative correlation (*p* < 0.05, *r* = 0.61). SR angiography with a HARP detector made it possible to identify impaired microcirculation in PE by means of its high spatial resolution and sensitivity.

## Abstract 2

Synchrotron radiation angiography with a HARP detector made it possible to evaluate impaired pulmonary microcirculation in pulmonary emphysema by means of high sensitivity.

## 1. Introduction   

In general, the quality of angiographic images is determined by three fundamental factors: (i) spatial resolution, (ii) time resolution and (iii) density resolution. However, modalities in which all three types of resolutions are satisfactorily provided at the same time have not been available. For example, small or minute changes in angiography have not been visualized *in vivo*. Transvenous angiography in pulmonary microcirculation is one of the subjects to be investigated in this regard.

Synchrotron radiation (SR) has been known to offer high spatial resolution owing to high photon density and high photon flux and parallel beam (Yamashita, 2001 ▶; Kobayashi *et al.*, 2004 ▶; Ikura *et al.*, 2004 ▶). Furthermore, synchrotron radiation angiography (SRA) can increase the time resolution owing to the use of a TV system that captures images at 30 frames s^−1^, which makes it possible to observe changes that take place in short intervals. We have been working to improve the effectiveness of SRA, by utilizing a high-gain avalanche rushing amorphous photoconductor (HARP) detector of extra-high sensitivity with moderate density resolution (Konishi *et al.*, 2011 ▶). The HARP detector developed by NHK (Japan Broadcasting Corporation) Science and Technology Research Laboratories (Tokyo, Japan) can theoretically be considered to be approximately 1000 times more sensitive than conventional CCD cameras (Konishi *et al.*, 2011 ▶; Tanioka *et al.*, 2003 ▶).

The purpose of this study was to investigate whether minute contrast changes of small perfusion could be identified with the high spatial and extra-high sensitivity with moderate density resolution and adequate time resolution provided by SRA equipped with a HARP detector. As a suitable pathological condition, peripheral perfusion in pulmonary emphysema (PE) was selected. PE is a disease involving decreased elasticity of the airway and alveolar wall destruction, as well as endothelial dysfunction of pulmonary microcirculation (Ito *et al.*, 2012 ▶). In this context, we hypothesized that detailed analysis of density using transvenous SRA with a HARP detector would make it possible to evaluate impaired microcirculation of PE by comparing histopathological changes such as the number of arterioles and degree of alveolar destruction *in vivo*.

## 2. Materials and methods   

### 2.1. Synchrotron radiation angiography   

SRA was performed at the Photon Factory-Accelerator Ring (PF-AR) at the High Energy Accelerator Research Organization in Tsukuba, Japan. Our SR was obtained from a 6.5 GeV electron beam. The polychromatic SR beam was monochromated by reflecting at 13° on a silicon crystal. The energy of the monochromatic X-rays was 33.3 keV with high photon density (10000 times that of conventional X-rays in clinical use provided by X-ray tubes) and a straight beam, which leads to high spatial resolution (Miyakawa *et al.*, 2010 ▶). The X-rays were converted to visible light on a fluorescent screen made of caesium iodide.

Angiographic images were obtained in high-resolution cineangiogram. The size of the visual field was 25 × 15 mm. The exposure time was fixed at 30 ms frame^−1^, and 30 frames s^−1^ of the angiographic images were captured using television camera technology (Miyakawa *et al.*, 2010 ▶). The rats were fixed at a distance of 4 cm from the fluorescent screen to protect the screen from unexpected motion of the rats.

### 2.2. HARP detector   

In the HARP detector, amorphous selenium is used for the photoelectric conversion film which converts light into an electrical signal (Tanioka *et al.*, 2003 ▶). When light is incident to the film, hole–electron pairs are generated, and accelerated in high electric fields. These are ionized by colliding and form new hole–electron pairs. The collision and ionization process is repeated, and the electric signal is multiplied. This is called the avalanche multiplication phenomenon (Tanioka *et al.*, 2003 ▶). The signal electric current output grows extremely large as a result of this multiplication phenomenon.

The number of pixels was 1920 × 1080 (Hi-vision standards) on the imaging system. The final spatial resolution in this SR system with the HARP detector was less than 25 µm.

The coupling between the phosphor (FOS J6679-01, Hamamatsu Photonics, Japan) and the HARP was lens coupling. There was no magnification. The contrast transfer function (CTF) was 33% and the resolution at the phosphor was 22 µm, which was the overall resolution as indicated by Hamamatsu Photonics. The pixel size of the HARP camera itself was 13 µm.

### 2.3. Animals   

The Committee on Animal Research at the University of Tsukuba approved the experimental protocols. The animals were cared for in accordance with the Guiding Principles for the Care and Use of Animals based on the Helsinki Declaration of 1964. Male Wistar rats (6 months; BW: 600 g; Charles River Japan, Yokohama, Japan) were divided into two groups according to with or without induction of pulmonary emphysema: group PE (*n* = 7), group C (control; *n* = 7).

### 2.4. Animal procedure   

Anesthesia was performed by inhalation of diethyl ether (Wako Chemicals, Japan), followed by intraperitoneal injection (50 mg kg^−1^) of pentobarbital (Somnopentyl R, Kyoritsu Seiyaku Corporation, Japan). A skin incision was made to expose the trachea. The frontal portion of the trachea was punctured by a 27-gauge needle for injection of porcine-derived elastase (200 U kg^−1^, Elastin Products, MO, USA) in group PE. Meanwhile, the same dose of normal saline was injected in group C.

### 2.5. Experimental protocol   

Three weeks after injection of elastase or saline through the trachea, the rats were involved in SRA and then sacrificed.

#### 2.5.1. Angiographic procedure   

After anesthesia, the right jugular vein was punctured by a 24-gauge needle and was cannulated with a plastic catheter sheath. A contrast material (32% non-ionic iodine) was administered by a programmed injector at 2 ml s^−1^ for 1 s.

#### 2.5.2. Storage of images   

Consecutive images were recorded as a digital movie, and the raw data were stored in a digital recorder and replayed using the Windows Media Video (WMV) format. WMV images were captured and converted into still images.

#### 2.5.3. Preparation for histopathology   

The lungs were removed and fixed with an intratracheal injection of 10% formalin solution under a constant pressure of exactly 20 cm H_2_O. These specimens were evaluated by histopathology [hematoxylin-eosin (HE) stain].

### 2.6. Evaluation of images   

#### 2.6.1. Locations for density measurement of contrast material   

**Figure 1:**
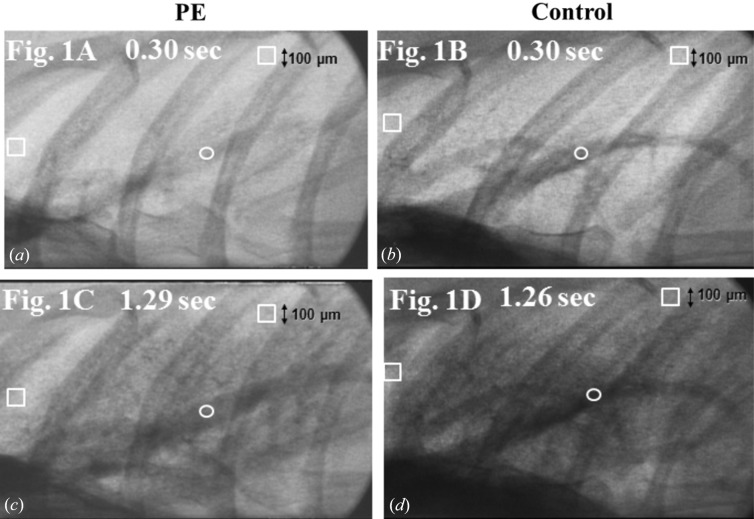
Examples of still images in each group. The peak density of the peripheral lung in the peripheral area in group PE (*c*) was lower than that in group C (*d*). The ROI in the peripheral lung (squares) and ROI in the PA (circles) are indicated.

The average densities of the contrast material were measured in the following specific locations. As for the peripheral area in the right upper lobe, the region of interest (ROI) was placed 1000 µm inside the visceral pleura at the apex avoiding the ribs and vessels. In the right lower lobe, the ROI was placed 1000 µm inside the visceral pleura at the first branch of the inferior pulmonary artery toward the right lower lobe in the craniocaudal axis avoiding ribs, vessels and diaphragm (Fig. 1 ▶). In the main pulmonary artery (PA), the ROI was placed at the first branch of the PA toward the right lower lobe (Fig. 1 ▶).

The data of the ROI were averaged by ten different ROIs in the described location. In the case of peripheral density, the data of the ROI in the upper and lower lobes were averaged.

#### 2.6.2. Measurement of density changes of contrast material   

The 256-grayscale value was employed using the graphic software *ImageJ* (NIH, USA). The grayscale value of each ROI represented the degree of X-ray absorption of iodine. High grayscale values indicated a high value of photon absorption by iodine.

#### 2.6.3. Mathematical analysis of the scatter plotting diagram   

**Figure 2:**
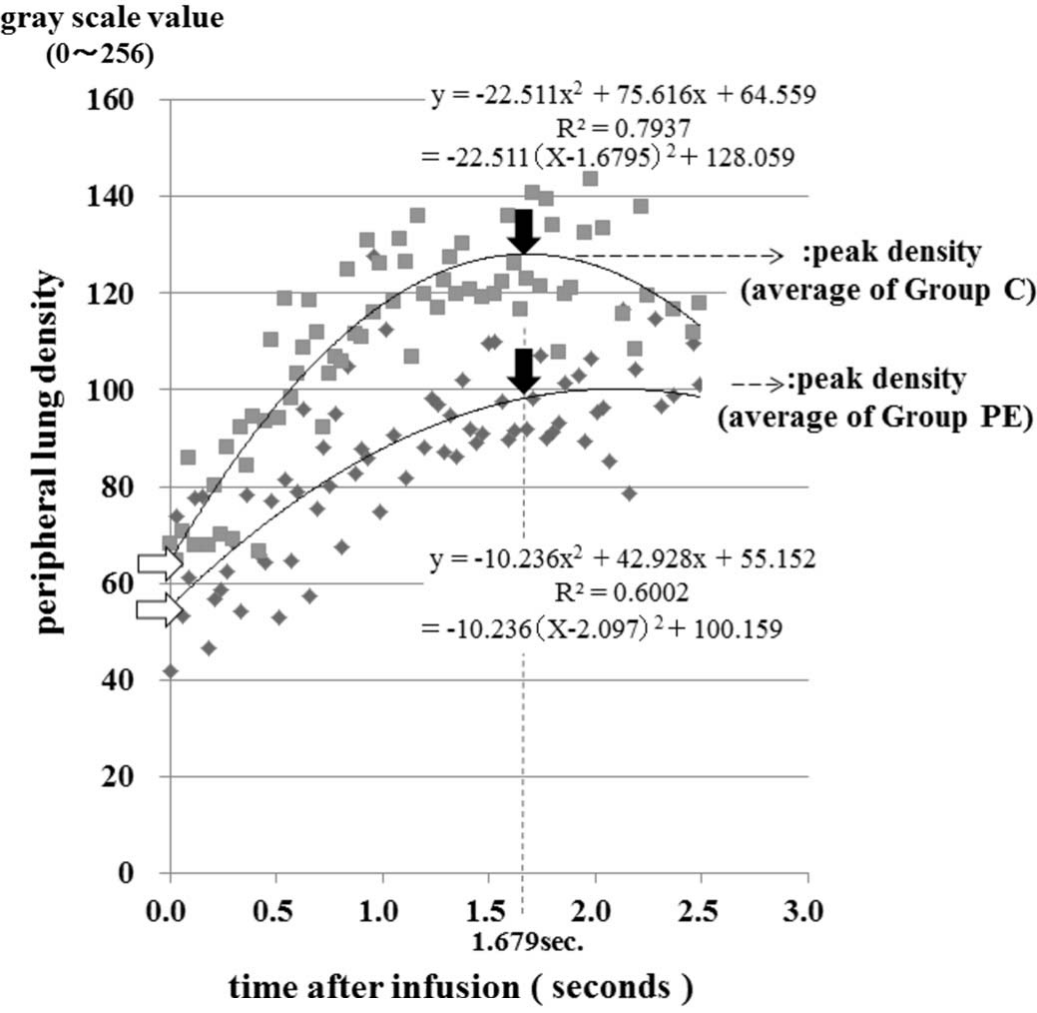
Each symbol represents the average density of seven rats in every instance of image taking. There was no significant difference statistically between groups C and PE at time zero (white arrows). The distribution of the symbols can apply to a quadratic function graph, and so the peak density can be determined. The peak density of group PE was significantly lower than that of group C (109 ± 10 *versus* 137 ± 11, *p* < 0.001), indicating a decrease in vascular volume in the peripheral area in PE. When compared at 1.7 s after initial infusion (black arrows), the density of group PE was also significantly lower than that of group C (97 ± 10 *versus* 129 ± 11, *p* < 0.001).

The abscissa (*X*-axis) was the time after infusion of contrast material in seconds. The ordinate (*Y*-axis) was the grayscale value of the ROIs with 8-bits provided by graphic software *ImageJ* (Fig. 2 ▶). The scatter graph up to 2.5 s could be approximated using a quadratic function formula and graph, which was determined by the least-squares method (Fig. 2 ▶).

**Figure 3:**
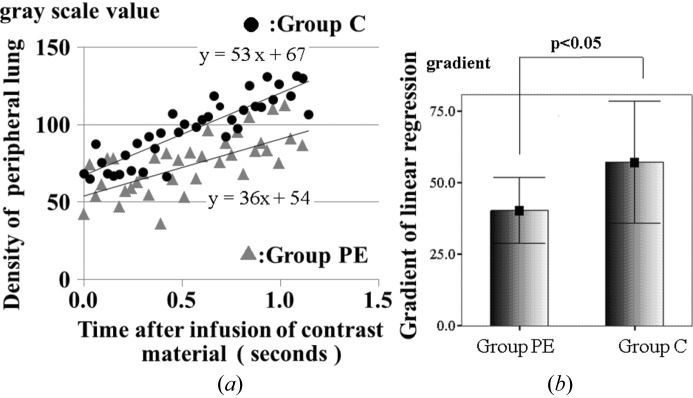
(*a*) In the initial 1.2 s, each plot of the average in the two groups can apply to the linear regression line. Each of the regression lines was confirmed as statistically significant (the *R* ^2^ value of groups PE and C were 0.447 and 0.779, respectively; *p* < 0.0001). (*b*). This figure was composed using seven slopes of linear regression lines in both groups. The slope of the linear regression lines in group PE (40.2 ± 11.5) was lower than that in group C (57.1 ± 21.3), a significant difference (*p* < 0.05). This indicated lower pulmonary blood flow in PE.

Furthermore, the slope of the simple linear regression line close to the peak was determined by the linear least-squares method in each rat (Fig. 3 ▶).

### 2.7. Parameters of analysis   

#### 2.7.1. Peak density   

The peak density of grayscale values in each quadratic function graph indicates the blood vessel volume at the peripheral lung area in each rat. Since the timing of the peak density is influenced by dilution or diffusion of contrast material in lung vessels, the peak time of group C (1.7 s from infusion) was chosen in order to compare the averaged peak density in both groups precisely (Fig. 2 ▶).

#### 2.7.2. Slope of linear regression line   

The slope of the linear regression line was obtained by least-squares methods from scatter plotting the peripheral lung density in each rat (Fig. 3 ▶). The slope of the linear regression line indicated the temporal increase of densities which may indicate blood flow in the peripheral lung.

#### 2.7.3. Correlation between slope and extent of PE   

The correlation between the slope of the linear regression line which was corrected by the main PA density and the degree of alveolar wall destruction was investigated. In other words, this correction was performed so that each slope of the linear regression line was divided by the slope of the linear regression line of the main PA density.

### 2.8. Histopathological analysis   

#### 2.8.1. Evaluation of alveolar wall   

**Figure 4:**
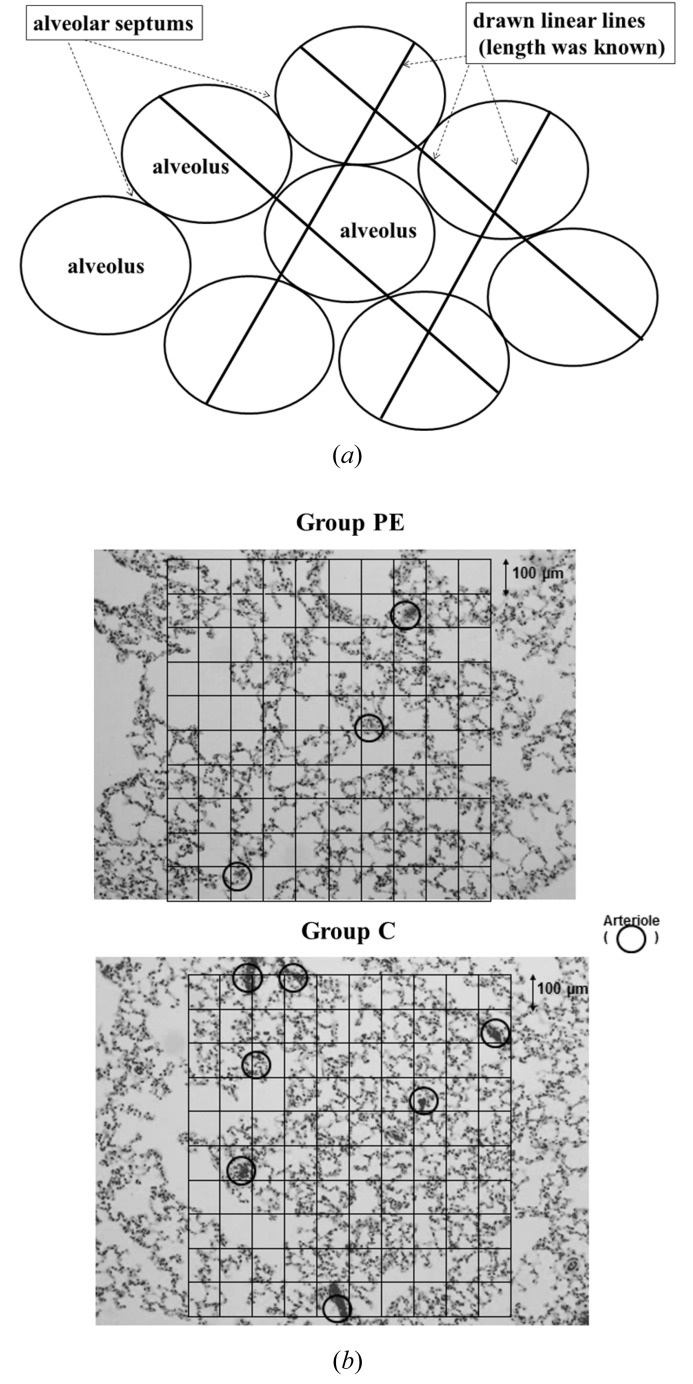
(*a*) An example diagram showing the concept of the point-counting method of Lm (mean linear intercept). Fixed-length linear lines were drawn randomly on alveoli. The length was divided by the number of points crossing alveolar septa giving the average of alveolar septum distances. (*b*) Typical pathological findings of lung from each group (100×) for counting of Lm and number of arteriole (mm^−2^). The top specimen showed the destruction of alveoli in pulmonary emphysema. The grid was covered by 1000 µm × 1000 µm. Lm was determined using this grid and crossing alveolar septum (details are given in §2). The arteriole is circled. The number of arteriole was also counted within a square of 1000 µm × 1000 µm. The number of arteriole was lower in PE.

The extent of emphysematous lesions was assessed by measuring the mean linear intercept using the method of Thurlbeck (Dunnill, 1962; Thurlbeck, 1967). For example, Fig. 4(*a*) ▶ shows the concept of the point-counting method of the mean linear intercept. Fixed-length linear lines were drawn randomly on alveoli. Then the length was divided by the number of points crossing the alveolar septa. This is the mean distance of the alveolar septum. The mean distance of the alveolar septum from numerous lines is usually used in evaluation of the alveolar density in chronic obstructive pulmonary disease, which is called the mean linear intercept (Lm) (Dunnill, 1962 ▶; Thurlbeck, 1967 ▶).

In this study, Lm was defined as the number calculated as follows: the length of all lines in the grid was divided by the total number of intercepts with alveolar septa in the alveolar area. This method can evaluate the average size of alveoli.

In more detail, ten randomly selected fields in the alveolar area were calculated. Using 22 horizontal and vertical straight lines (eleven-by-eleven) in a grid, the points in the alveolar walls crossing the lines were counted using HE staining. The magnification was 100× in light microscopy. The length of each line was 100 µm (Fig. 4*b* ▶). The data from ten grids were averaged (Yamada *et al.*, 2004 ▶).

#### 2.8.2. Evaluation of the microvasculature   

The same ten randomly selected fields that were previously used in the evaluation of Lm were employed (Fig. 4*b* ▶); the magnification was the same, 100×, using light microscopy. The number of arterioles was evaluated by counting the arterioles within or crossing the border line of a 1 mm^2^ square in HE stained specimens. Arterioles were defined as arteries of more than 50 µm and less than 200 µm in diameter in this study. Furthermore, the number of arterioles was averaged in ten fields. The number of arterioles was considered to be representative of the density of the microvasculature. The average numbers of arterioles in the two groups were compared.

#### 2.8.3. Statistics   

All variables were expressed as mean ± standard deviation. An unpaired Student’s t-test was employed for comparison. Simple linear regression analysis was performed for investigating correlations. A difference of *p* < 0.05 was considered significant. Statistics software *SPSS* (SPSS Inc., Chicago, IL, USA) was used.

## 3. Results   

### 3.1. Angiographic findings   

A scatter plotting diagram was made showing time and the average density of the peripheral lung [Fig. 2 ▶ and Fig. 3(*a*) ▶]. The symbols represent the averages of contrast material density in the peripheral lung of the two groups from the initiation of the rapid infusion of contrast material.

After infusion of contrast material, the density increased constantly until around 1.2 s, then the density reached a peak around 1.7 s. After that, the density began to decrease over 2.5 s (Fig. 2 ▶). It was possible to determine the peak density by applying a quadratic function. The average of the densities of group PE was significantly lower than that of group C at the peak in each group (109 ± 10 *versus* 137 ± 11, *p* < 0.01). Since the time of the peak density in group PE was relatively later than that in group C, the time of the peak density of the control group was chosen for comparison of the two groups at one particular moment. When compared at 1.7 s after initial infusion (the peak of the control group), the density of group PE was also significantly lower than that of group C (97 ± 10 *versus* 129 ± 11, *p* < 0.001) (Fig. 2 ▶).

The scatter plotting diagram could be expressed as a linear regression line between 0 and 1.2 s with significant correlation coefficients. The *R* ^2^ values of groups PE and C were 0.447 and 0.779 (*p* < 0.0001), respectively (Fig. 3*a* ▶).

The slope of each linear regression line of the peripheral lung in both groups was compared (Fig. 3*b* ▶). The average of the slope of the linear regression line in group PE was significantly lower than that in group C (40.2 ± 11.5 *versus* 57.1 ± 21.3, *p* < 0.05).

### 3.2. Histopathological findings (Fig. 4*b*)   

Comparison of Lm and the number of arterioles per mm^2^ between groups PE and C was performed. The average for Lm was 178 ± 20 µm in group PE and 126 ± 7 µm in group C (*p* < 0.001) (Fig. 4*b* ▶). A higher value of Lm meant an enlarged size of the alveoli owing to destruction of the alveoli.

The average number of arterioles per square millimetre in each group was as follows: group PE, 2.4 ± 0.3 mm^−2^; group C, 4.8 ± 0.3 mm^−2^ (*p* < 0.001). The number of arterioles was significantly lower in group PE. These results on microvasculature possibly involved the apparent difference of peripheral perfusion between the two groups.

### 3.3. Correlation between the slope of linear regression line and alveolar destruction (Lm) in PE (Fig. 5)   

The average of the slope of the linear regression line divided by the slope of PA density was significantly lower in group PE than in group C (group PE: 0.53 ± 0.16; group C: 0.71 ± 0.19, *p* < 0.05).

**Figure 5:**
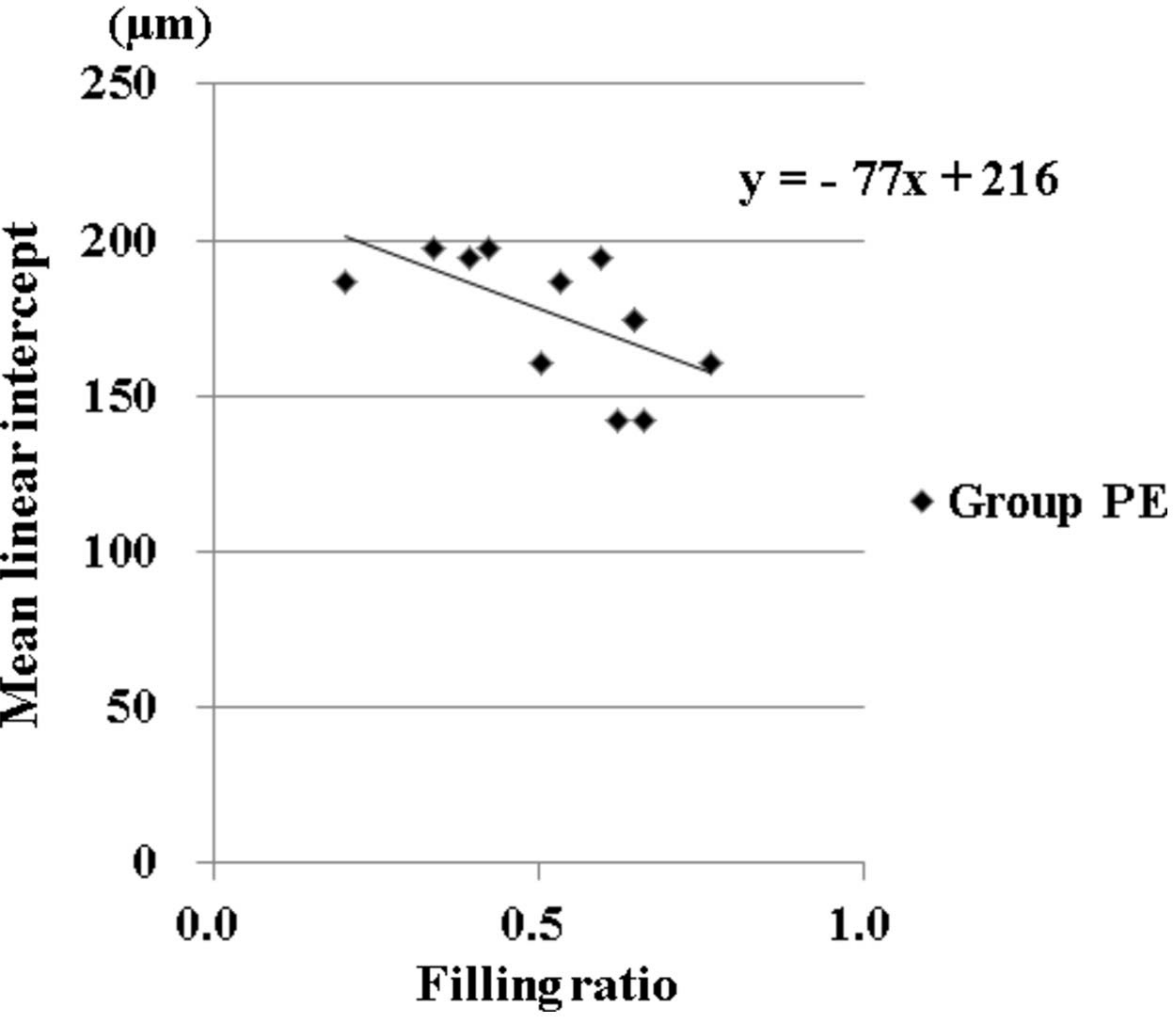
Correlation between the slope of the linear regression line and Lm in group PE showing significant negative correlation (*r* = 0.61, *p* < 0.05). The slope employed in this investigation is derived from the slope in the peripheral area divided by the slope in the main pulmonary artery in each rat in order to adjust the basic supply to pulmonary circulation. The low slope of the linear regression line represents decreased pulmonary blood flow in peripheral circulation in PE, which has never been obtained except for SRA with the HARP detector.

The correlation between the slope of the linear regression line and the Lm of each rat in group PE is shown as a scatter plotting diagram in Fig. 5 ▶. The symbols are expressed as a linear regression line with a significant correlation coefficient (*R* value: 0.61, *p* < 0.05). Rats with a lower slope of linear regression line indicated more severe destruction of alveoli in group PE.

## 4. Discussion   

Pulmonary emphysema is a disease characterized by destruction of alveoli with decrease of microvasculature. However, no modality can evaluate correctly both alveolar destruction and impairment of microvasculature at the same time. In our experimental models, both alveoli and arterioles were decreased and confirmed by histopathology in this study. Furthermore, these minute changes can evaluate effectively by use of SRA with a highly sensitive HARP detector. The peak density of contrast material in the peripheral lung of PE was significantly decreased compared with the control, which indicates the loss of blood volume in pulmonary microvasculature in PE. The slope of the linear regression line indicates a temporal increase of densities representing an increase rate of blood flow in the peripheral lung. Therefore, angiographic findings in this system may indicate not only anatomical change but also functional change in pulmonary microcirculation. On the other hand, Lm represents anatomical destruction of alveoli by inflammation in PE. Thus, Lm indicates the extent of PE. In this study there was significant negative correlation between the slope of the regression line and Lm in Fig. 5 ▶, which represents the meaningful relation of decrease of blood flow and the extent of PE in the peripheral lung area. This result was acceptable, because an impaired endothelial function has been found in small pulmonary arteries from patients with PE (Peinado *et al.*, 1998 ▶; Kasahara *et al.*, 2001 ▶). These pathophysiological vascular changes might explain the negative correlation between the rate of blood flow and the extent of PE.

Changes in pulmonary microcirculation were successfully analyzed because of the following radiographic properties: (i) high spatial resolution, (ii) high sensitivity and (iii) adequate time resolution. The spatial resolution of this system is less than 25 µm (Konishi *et al.*, 2011 ▶). High spatial resolution and high sensitivity were able to contribute to the significant linear regression lines and their significant difference in slopes of the linear regression line. It is mainly derived from SR with the HARP detector, which can amplify the dynamic range of the grayscale by approximately 1000 times compared with conventional CCD cameras (Konishi *et al.*, 2011 ▶; Tanioka *et al.*, 2003 ▶).

Although the density resolution of a CCD camera is not inferior to a HARP detector, an extraordinary increase in the sensitivity in the HARP detector makes diluted contrast material visible even in the peripheral lung. This SR angiographic system with the HARP detector is so promising because it possesses several advantages simultaneously: sensitivity, spatial resolution and time resolution, which were not used previously (Mori *et al.*, 1994 ▶, 1996 ▶; Takeshita *et al.*, 1997 ▶). We have been working to improve the effectiveness of high-resolution angiography using SR *in vivo* for the past ten years (Matsushita *et al.*, 2008 ▶), and it is now possible to visualize down to a coronary artery spasm of ∼50 µm using a HARP detector (unpublished data).

The density resolution of 8-bits in our experiment made it possible to analyze this visualized contrast material by amplifying the sensitivity using the HARP detector. In short, the role of the HARP detector is to visualize the diluted contrast agent with the extra high function of amplification. As a consequence of this visualization, it became possible to accurately measure the minute difference in density easily, even in 8-bits in this system.

In addition, the role of synchrotron radiation is to increase the spatial resolution. Sufficient spatial resolution of this system (using SR), excellent high-performance amplification (using the HARP detector) and adequate time resolution (30 frames s^−1^) made it possible to measure the minute density change in a lung, reflecting the change of peripheral arterial blood flow in the rat model of pulmonary emphysema.

Since no modality had been available to measure minute changes of pulmonary circulation, it has been desirable to develop new systems that would enable analysis of the impaired microcirculation of the peripheral lung in PE (Suhonen *et al.*, 2008 ▶; Schwenke *et al.*, 2007 ▶, 2008 ▶). In the case of CT angiography, minute changes of small perfusion cannot be recognized mainly due to limited time and density resolution (Koh *et al.*, 2003 ▶). In the case of lung perfusion scintigraphy, it is not enough to evaluate microcirculation of the alveolar area, owing to limitations of spatial resolution (Fink *et al.*, 2003 ▶; Molinari *et al.*, 2006 ▶). On the other hand, more sensitive devices for density resolution, such as high-resolution three-dimensional magnetic resonance angiography (MRA), have been developed over the past decade (Molinari *et al.*, 2006 ▶; Kennedy *et al.*, 2006 ▶). However, the density resolution of MRA, contrary to expectation, has not been satisfactory (Molinari *et al.*, 2006 ▶). The spatial and time resolutions of MRA are inferior to traditional angiography (Kennedy *et al.*, 2006 ▶). Compared with the above-mentioned modalities, our transvenous SRA can be seen as providing a possible solution to this problem.

Pulmonary emphysema is one of the most common reasons for respiratory failure, and is ranked as the fourth most common cause of death in developed countries (Fernandes *et al.*, 2011 ▶). Recently, it has been reported that apoptosis of vascular endothelial cells is closely related to the pathogenesis of PE (Siafakas *et al.*, 2007 ▶). Also, in the past decade, it has been recognized that endothelial dysfunction and impaired pulmonary circulation might play important roles in the pathogenesis of PE (Magee *et al.*, 1988 ▶; Barberà *et al.*, 2001 ▶, 2003 ▶). The lowered slope of the linear regression line obtained in this study might have been influenced not only by structural alveolar change but also by the impaired functional change of microvasculature due to such factors as decreased endothelial nitric oxide synthase (eNOS) expression in PE (Ito *et al.*, 2012 ▶; Peinado *et al.*, 1998 ▶; Kasahara *et al.*, 2001 ▶). eNOS is one of the most important factors for vasodilatation. In this regard, the evaluation of minute changes of impaired microcirculation in the peripheral lung may contribute to understanding the current situation of pathophysiology from the vasculature in PE. Interestingly, statins have a protective effect on alveolar and capillary apoptosis in PE (Takahashi *et al.*, 2008 ▶; Dimmeler *et al.*, 2001 ▶), which may provide the prevention of death from PE. It is important to detect impaired pulmonary perfusion in non-invasive radiologic studies in order to initiate angio-protective therapy including statins in PE (Takahashi *et al.*, 2008 ▶; Dimmeler *et al.*, 2001 ▶; Llevadot *et al.*, 2001 ▶; Lee *et al.*, 2005 ▶) before advancement of the disease. In this context it can be expected that accurate evaluation of pulmonary microcirculation by the use of highly sensitive SRA will accelerate early treatment of microvascular impairment and ventilation-perfusion mismatch, leading to the preservation of quality of life and improvement of prognosis in patients with PE.

## 5. Conclusion   

SR angiography with a HARP detector has made it possible to evaluate impaired pulmonary microcirculation in PE by means of high sensitivity along with high spatial resolution and time resolution.
